# Supplementary material for: The optimal warming strategy to reduce perioperative hypothermia: A prospective randomized non-blinded clinical trial
Source: PLoS One. 2025 Jun 12;20(6):e0325954. doi: 10.1371/journal.pone.0325954 (PMC12161556; doi:10.1371/journal.pone.0325954)
Supplement: S2 Appendix — (DOCX) [file pone.0325954.s002.docx]

**Mixed model for Randomized Block Design**

In this study, patients were randomly assigned between the four rewarming groups within 10 surgery blocks. The AUC values were computed for every patient assigned in the study. The primary objective of this study was to compare the AUC values between the 4 arms. To achieve this, mixed model for continuous outcomes (AUC) was used with the arms as the fixed effect variable and the surgery units as random effect variable. A description of the model and the effects of interest as used in this study is provided below:

Let **y*_as_*** denote the AUC values from the patients that were assigned to arm ***a*** in surgery ***s***, the equation for the model is

**y_as_ = µ + τ_a_ + *b_s_ + e_as_***

where

**a** = 1, 2, 3, 4 arms

**s** = 1, 2, … 10 surgery units

**µ** and **τ_a_** = are fixed effect parameters with **µ** representing the overall mean, and

**µ_1_ =** **µ + τ_1_, µ_2_ =** **µ + τ_2_, µ_3_ =** **µ + τ_3_, µ_4_ =** **µ + τ_4_**

***b_s_* =** is the random effect associated with the s^th^ surgery unit

**e_as_** = is the random error associated with the patient in surgery ***s***, and assigned to arm, ***a***.

The SAS code below uses the above mixed model, to answer the specific research questions from this study:

/********************************************************/

********** Mixed Model for Randomized Block Design *****;

* gp = the grouping variable containing the 4 arms

strat = the blocking variable respresenting the 10 surgery units

lg1 = log (AUC + 1). This is the outcome ;

/*******************************************************/

**proc** **mixed** data=df4;

class gp strat;

model lg1=gp ;

random strat;

lsmeans gp / pdiff; *Test for all pairwise differences between groups;

/* Calculates the mean LG1 for each group */

estimate '1 mean' intercept **1** gp **1** **0** **0** **0**;

estimate '2 mean' intercept **1** gp **0** **1** **0** **0**;

estimate '3 mean' intercept **1** gp **0** **0** **1** **0**;

estimate '4 mean' intercept **1** gp **0** **0** **0** **1**;

/* Calculates the differences in LG1 between groups */

estimate '1 vs 4' gp **1** **0** **0** -**1**;

estimate '1 vs 3' gp **1** **0** -**1** **0**;

estimate '1 vs 2' gp **1** -**1**;

estimate '2 vs 3' gp **0** **1** -**1** **0**;

estimate '2 vs 4' gp **0** **1** **0** -**1**;

estimate '3 vs 4' gp **0** **0** **1** -**1**;

estimate '12 vs 34' gp **1** **1** -**1** -**1**;

estimate '13 vs 24' gp **1** -**1** **1** -**1**;

**run**;
