# Supplementary material for: The optimal warming strategy to reduce perioperative hypothermia: A prospective randomized non-blinded clinical trial
Source: PLoS One. 2025 Jun 12;20(6):e0325954. doi: 10.1371/journal.pone.0325954 (PMC12161556; doi:10.1371/journal.pone.0325954)
Supplement: Attachment — Submitted filename: R1988d_PLOS One - response to reviewers.docx [file pone.0325954.s004.docx]

Dear Dr. Desai,

Thank you for submitting your manuscript to PLOS ONE. After careful consideration, we have decided that your manuscript does not meet our criteria for publication and must therefore be rejected.

I am sorry that we cannot be more positive on this occasion, but hope that you appreciate the reasons for this decision.

Kind regards,

Steven E. Wolf, MD
Academic Editor
PLOS ONE

Additional Editor Comments:

Academic Editor - Thank you for submitting your paper to us for review. I sent it to seven distinguished referees for comment and decision of whom three agreed to review; you will see these below. Unfortunately, the referees found some significant issues with the paper, and could not recommend publication. Most of this has to do with the statistical rendering of the results, which has some holes in it as described. For my part, I feel this is a reasonably well done study with supportable conclusions, however, the statistical issues have to be addressed fully. This might result in some significant changes in the conclusions, so the final decision was for reject but with hearty encouragement to address these issues and resubmit. It does not really fit as a major revision since the conclusions have a reasonable probability of significant change. I hope you will consider this solution, and I look forward to seeing it come back.


Reviewers' comments:

Reviewer's Responses to Questions

**Comments to the Author**

1. Is the manuscript technically sound, and do the data support the conclusions?

The manuscript must describe a technically sound piece of scientific research with data that supports the conclusions. Experiments must have been conducted rigorously, with appropriate controls, replication, and sample sizes. The conclusions must be drawn appropriately based on the data presented.

Reviewer #1: Yes

Reviewer #2: Partly

Reviewer #3: No

2. Has the statistical analysis been performed appropriately and rigorously?

Reviewer #1: Yes

Reviewer #2: I Don't Know

Reviewer #3: No

3. Have the authors made all data underlying the findings in their manuscript fully available?

The [PLOS Data policy](http://secure-web.cisco.com/1QhVL8NXuEpSb4ZBpFTF-sj2qy6laNuC_d85Bd8oTeXNhcn5y42uYKK2qG97y4wwAD6uM_Q5qgP5t5sUtgvB66x96uP3isfDA5zUwoINATKOCivkw94zSd1-wwRFhTL6elXjns1MVqWjAt3fCrcgHUKYxJItknEp3dvM-6t_Py5l3Sc9Ii2qyovXmGng0uAt8nXVeURKo_rw-5UsO8sA2_V3kFANbGZeytWXThmdy-4tYYmapAlW7HvibNlMVj0JuSRmqPOQCJZwre_7lBDkM4ZBzL39tylSq_uZEelTcLEepXCafJnD6DYzQZcAMvDG8/http%3A%2F%2Fwww.plosone.org%2Fstatic%2Fpolicies.action%23sharing) requires authors to make all data underlying the findings described in their manuscript fully available without restriction, with rare exception (please refer to the Data Availability Statement in the manuscript PDF file). The data should be provided as part of the manuscript or its supporting information, or deposited to a public repository. For example, in addition to summary statistics, the data points behind means, medians and variance measures should be available. If there are restrictions on publicly sharing data—e.g. participant privacy or use of data from a third party—those must be specified.

Reviewer #1: Yes

Reviewer #2: Yes

Reviewer #3: No

**Authors’ response re #3: We have completely revised the statistical approach and in addition to previously submitted data, we have attached the mixed model and the randomization table to the new submission.**

4. Is the manuscript presented in an intelligible fashion and written in standard English?

PLOS ONE does not copyedit accepted manuscripts, so the language in submitted articles must be clear, correct, and unambiguous. Any typographical or grammatical errors should be corrected at revision, so please note any specific errors here.

Reviewer #1: Yes

Reviewer #2: Yes

Reviewer #3: No

5. Review Comments to the Author

Reviewer #1

1. Desai and colleagues investigated the topic of perioperative hypothermia through a combination of pre- and intra-operative forced air warming and conductive warming methods. They concluded that intra-operative conductive warming (underbody plus over body cover) was superior to forced air warming when prewarming was not possible, and that intra-operative forced air warming alone is inferior to the other methods tested. However, if patients undergo prewarming then both strategies are equivalent.

The manuscript reads well, references relevant literature, and addresses a common perioperative quality metric and patient safety concern. No major weaknesses noted. One minor point is that my brief literature review revealed a couple recent systematic reviews on prevention and management of perioperative hypothermia in adult elective surgical patients, which found forced air warming to be effective and may warrant comment in the author’s discussion (Simegn G. Ann Med Surg 2021 Nov 14:72:103059 and Moola S. JBL Libr Syst Rev 2010;8(19):752-792).

**Authors’ response: Thank you for these suggestions. We have now cited two extensive reviews that are highly relevant to the discussion:**

**Madrid E, Urrutia G, Roque i Figuls M, Pardo-Hernandez H, Campos JM, Paniagua P, et al. Active body surface warming systems for preventing complications caused by inadvertent perioperative hypothermia in adults. The Cochrane database of systematic reviews. 2016;4(4):CD009016. Epub 20160421. doi: 10.1002/14651858.CD009016.pub2. PubMed PMID: 27098439; PubMed Central PMCID: PMCPMC8687605.**

**Moola S and Lockwood C. The effectiveness of strategies for the management and/or prevention of hypothermia within the adult perioperative environment: systematic review. JBI Libr Syst Rev 2010; 8: 752-792. DOI: 10.11124/01938924-201008190-00001**

The authors eloquently address how their results contrast published studies that inconclusively reported the effects of conductive warming. The study design and thorough data collection adds significant value to published work on this topic by collecting temperature measurements throughout the intra-operative time frame and using the same temperature settings regardless of device used.

I recommend publishing.

2. Areas for improvement
a. Major issues: none
b. Minor issues: consider reviewing systematic reviews noted above for potential additional discussion points

Reviewer #2:

Dear Authors,
This is a nice and interesting study. In this study, the authors have compared the effects of prewarming and intraoperative warming modes (with forced-air warming (FAW) and conductive warming (CW) methods) on the incidence and duration of hypothermia.
In this study, unlike other research, perioperative hypothermia has been evaluated by measuring the area under the curve (AUC)< 36°C.
The research contains some limitations. Failure to ensure homogeneity of patient and surgical methods and protective measures against hypothermia should be presented in the limitation section.
The statistics of the article should be re-evaluated.
I reviewed this article and have a few editing suggestions.
Sincerely
Suggestions:
1. Materials and Methods/Participants. In this prospective randomised study has evaluated patients who elective surgery under general anaesthesia. However, homogeneity could not be achieved with surgery types (major surgery or minor surgery). Were patients using medications that would affect body temperature changes excluded? The research contains some limitations. Failure to ensure homogeneity of patient and surgical methods and protective measures against hypothermia should be presented in the limitation section.

**Authors’ response: We thank the reviewer for this advice. We have added the full randomization table (“S1 Appendix - Randomization chart.xlsx”) with the types of elective surgeries enrolled and have added a comment about homogeneity in the discussion.**

2. Materials and Methods/ Randomization. (line 156-158) It is written, “Patients were contacted twice postoperatively, at 24 hours and then 30 days to assess for any complications not recorded in the electronic medical record.”. These data are not available between the primary and secondary outcoms of the study.

**Authors’ response: We have added a table to report the post-operative complications and events as supplementary material – “S3 Appendix - Secondary outcomes and complications.xlsx”.**

3. Materials and Methods/ Procedures: When was the first temperature measurement made? Please explain. Please detail other precautions to prevent perioperative temperature decrease (e.g. warming of intravenous and infusion fluids, etc.). Were the patients' basal temperatures evaluated before surgery?

**Authors’ response: We thank the reviewer for this advice. We have added this to the methods section, subsection on ‘Procedures’.**

4. “In Table 2: Regression analyses of the hypothermia AUC for each trial arm”. The purpose of the comparison between groups' linear regression test should be explained in the result section.

**Authors’ response: all tables have been completely reworked in line with the new analysis and results.**

Reviewer #3

The authors reported about the results of prospective, non-blinded, randomized single center clinical trial with a 4 arm parallel group design to explore the effect of two different ways to warming strategies (preoperative and intraoperative) with no application of preoperative warming, or FAW or CW. They concluded that intraoperative FAW alone is an inferior warming strategy compared to three other methods: CW/CW, FAW/FAW, and NAPW/CW and thus patients may benefit from the routine implementation of active warming for at least 30 minutes preoperatively. When prewarming is not feasible, intraoperative CW is a superior warming strategy compared to FAW.

In general, this is a nice study but due to major discrepancies between planning and conduct as well as planning errors, it appears to have unsurmountable weaknesses and result in unreproducible research.

To be more specific:

**Authors’ response: We thank the reviewer for the thorough review. As to the hypotheses, they were merely reworded in the original manuscript for clarity (shown in table below). However, we did a poor job of explaining that, in reality, there was only one leading hypothesis, namely that there would be a significant difference in the mean area under the curve (AUC) of temperature below 36℃ during the intraoperative period between the four combinations of warming strategies. We have now clearly stated that in the last paragraph of the introduction and have removed all references to three separate hypotheses.**

| Hypotheses as stated in the protocol | Hypotheses as stated in the original manuscript |
| --- | --- |
| 1. Active warming with CW will be non-inferior in preventing hypothermia when compared to active warming with FAW. 2. Active preoperative warming with CW or FAW will lead to a reduction in intraoperative hypothermia when compared with only intraoperative warming with CW or FAW. 3. Active preoperative warming combined with intraoperative warming using CW is a superior strategy compared to only intra-operative warming with the CW, intraoperative warming with FAW only, or pre-operative and intra-operative warming with FAW. | 1. Active intraoperative warming with CW is non-inferior to FAW in preventing intraoperative hypothermia. 2. Actively prewarming for at least 30 minutes with either CW or FAW reduces intraoperative hypothermia compared to no active prewarming (NAPW) with intraoperative CW or FAW, respectively. 3. Actively prewarming with CW combined with intraoperative CW is superior to NAPW with intraoperative CW or FAW, and is superior to prewarming and intraoperative warming with FAW. |

The author referred to 3 hypothesis (the first one differs from the initial protocol and serves as sample size justification) which may be formalized as

H01: {\mu_(CW,CW) = {\mu_( NAPW,CW)} or {\mu_(CW,CW) = {\mu_( NAPW, FAW)} or {\mu_(CW,CW) = {\mu_( FAW/FAW)}

H02: {\mu^2_(CW or FAW, CW or FAW)= {\mu^2_( NAPW, CW or FAW)}

H03: |{\mu_(CW,CW) = {\mu_( FAW/FAW)} | ≤ ∂ =1

These specific set of family of hypotheses cause a multiple testing problem, which has to be considered in the sample size justification, and analysis strategy. Moreover, the coherence between the statistical analysis model and the stratification factors of the randomization has to be considered. Both aspects are not fulfilled and thus the results and conclusion might change completely.

L150ff: Description of the randomization process: The randomization algorithm and software for generation of the randomization list is not given. The total of 10 stratification groups are not clear. In fact, the number of 10 stratification factors lead to an expected number of 18.4 patients per stratification factor level which are allocated to 4 treatment groups. This offers high predictability and thus allocation bias, in particular in a open study, depending on the randomization algorithm. Further samples size obviously does not result in a 1:1:1:1 allocation ratio.
Concerning the implementation it is not detailed, how concealment of the randomization process was achieved.

**Authors’ response: We have added the full randomization table (“S1 Appendix - Randomization chart.xlsx”) which makes the 10 stratification groups clear, and have explained in the body of the text that we used the random number function in Excel to create the table. The method of randomization concealment is also explained.**

L182: The sample size justification could not be followed for various reasons and may be wrong, resulting in a not well powered study.
First the proposed inclusion of the hypotheses, by which no adjustment of the significance level is needed, applies not to H02, as the endpoint is measured on different times. Further although the third part of H01 implies H03, the converse is not true. And of note the analysis population of H03 differs from H01. So sample size justification for H01 does not necessarily apply to the other ones.
The effect size stated for H01 declaring a difference of 1 as clinically relevant with the ST of 0.3 to 0.6 results in huge effect sizes of 1.67 to 3.3 resulting in rather small (2-7) sample sizes. This explains that the sample size justification could not be followed.
And, it is rather critical, to argue, that the non-inferiority margin has the same value, than the expected clinically relevant difference.
Calculation of adjustment for dropout rate is 4x40/0.85=188.23~189
**Authors’ response: a new sample size calculation was done based on a null hypothesis of equal means among the 4 treatment arms. This is clearly explained in the methods section. It yielded a slightly larger sample size of 216 for the study.**

L199: Measurement method of temperature should be given. Note according to the protocol different methods are used for pre/post and intraoperative measurement.

**Authors’ response: the temperature measurement method is clearly stated in the ‘Procedures’ section under ‘Methods’.**

L214: Statistical analysis is only partly according to the protocol and even there not appropriate.
The Shapiro Wilk's pretest needs to be included in a multiple testing decision rule framework. Corresponding type one error adjustment is needed. This test is superfluous in RCT's.
The statistical test on the main hypothesis does not follow the planned one. The statistical model deviates from the one used for sample size justification and the randomization process. Thus the power requirement is not given.
By the way, the stratification factor surgical approach has 5 and not 2 levels.
**Authors’ response: the statistical approach was completely redone and this is explained in detail in the new manuscript, as well as in ‘S2 Appendix - Mixed model.docx’.**

L232: Enrollment period is missing.

**Authors’ response: the enrollment period is now clearly stated under ‘Ethics approval and registration’ under ‘Methods’ (towards the end of the paragraph).**

L240: Statistical test in table 1 should be deleted as test on baseline characteristics are not meaningful in RCTs.

**Authors’ response: Table 1 has been completely redone.**

L243: Test results with application of the primary hypotheses should be stated explicitly.

**Authors’ response: this has been done in the new manuscript. The primary hypothesis and test results are stated clearly.**

L253: p-values need to be adjusted for multiple comparisons. Note regression coefficient do correspond to differences of means. These descriptive measures are informative here.
**Authors’ response: the statistical approach was completely redone and this is explained in detail in the new manuscript, as well as in ‘S2 Appendix - Mixed model.docx’.**

L350: Limitation section with discussion on the impact of non-blinding, allocation bias, missing observations etc on the study results need to be included.

**Authors’ response: this has been done. We have an expanded and updated limitations section under ‘Discussion’: ‘Methodological considerations and limitations.’**
