# Supplementary material for: The optimal warming strategy to reduce perioperative hypothermia: A prospective randomized non-blinded clinical trial
Source: PLoS One. 2025 Jun 12;20(6):e0325954. doi: 10.1371/journal.pone.0325954 (PMC12161556; doi:10.1371/journal.pone.0325954)
Supplement: Attachment — Submitted filename: Desai et al response to reviewers - R1.docx [file pone.0325954.s005.docx]

The optimal warming strategy to reduce perioperative hypothermia: a prospective randomized non-blinded clinical trial

**Manuscript Number:** PONE-D-24-44335

| **Reviewer #1:** | |
| --- | --- |
| **Comments** | **Author Responses** |
| GENERAL  As a reviewer, I commend the authors for conducting a well-designed study to investigate the impact of different warming strategies on intraoperative hypothermia. The use of a randomized controlled trial and the objective measure of AUC below 36°C are the strengths of the study. | We thank the reviewer for their detailed assessment of our manuscript. |
| SPECIFIC  However, a few areas could be further explored: Sample Size: Consider whether the sample size was adequate to detect clinically significant differences, especially for secondary outcomes, as it was mentioned in the study limitations. | Sample size was discussed as a limitation in the methodological considerations and limitations section. This discussion was expanded to include the following sentence:  “The study had the following limitations. Study enrollments fell short of the 216 minimum needed to detect a 1.0 °C*hour difference in AUC with 80% power. The enrolled subjects were predominantly female (80%), reflecting the patient population presenting for abdominal, gynecologic, breast, plastic, and urological surgery in our hospital. Homogeneity by type of surgery was not perfect in the 10 randomization groups, as there were more patients in the abdominal and the gynecologic surgery groups than the other groups (breast, plastics/reconstructive, and urological) – see S1 Appendix - Randomization chart. *Therefore, findings from the current study would benefit from confirmation in a larger clinical trial*.” |
| Heterogeneity in Surgical Procedures: The inclusion of various surgical procedures might have influenced the results. A more homogeneous patient population could provide clearer insights. | We acknowledge that the wide range of surgical procedures may limit the interpretation of data as it decreases standardization, including a variety of exposed surgical sites which may vary in rate of heat loss. However, as noted in the manuscript, peri-operative warming is used across a wide range of surgical procedures, and studying different procedures may more closely reflect real-world use. This may make the study more interpretable across specialties, given that there were minimal limitations to inclusion based on surgical procedure type. Moreover, surgery type was included as a blocking factor in the randomization schedule to avoid imbalance across groups. |
| Non-Standardized Blanket Use: The lack of standardization in blanket use in the NAPW arms is a limitation that could affect the results | We acknowledge that the implementation of blanket use was not standardized in the NAPW group. This may introduce variability, as a patient wearing a cotton blanket may retain more heat than a patient without a cotton blanket. Patients undergoing surgery may have differences in baseline thermal comfort based on various demographic and clinical characteristics (e.g. incidence of hot flushes of peri-menopause, metabolic health, known or unknown anemia, etc). Whereas the NAPW blanket intervention was not standardized, it was the clinical standard to allow patients thermal comfort in the NAPW arms, and thus they were permitted to use or defer cotton blankets to obtain thermal comfort. Therefore, this methodology reflects real-world practice, which facilitates the generalization of results. The non-standardized use blankets in the NAPW arms is described in the methodological considerations and limitations section:  “Warmed cotton blankets were applied to patients in the NAPW arms in a non-standardized fashion, i.e. only patients who wanted them were covered with blankets, and the temperature of the blankets was not recorded.” |
| The abstract is well-structured and concise. However, consider further condensing the methods section by focusing on the key aspects of the study design, such as the randomization process, intervention details, and primary outcome measure. | The abstract was edited with a focus on further condensing content. |
| Revise the whole manuscript for any grammatical and language mistakes | Line-item edits recommended by the reviewer have been made where appropriate, or the text has been edited for the purpose of clarification. We are grateful for the reviewer’s attention to detail. Additional edits were made in a full review of the manuscript and are tracked in the highlighted version of the manuscript. |
| Line 31: Consider rephrasing "We conducted a prospective observational study" to "We conducted a prospective randomized controlled trial." | Modified as suggested. |
| Line 36: The equation for calculating AUC could be simplified or presented in a more concise format. | We added a description of T1 and T2 to the formula based on feedback from Reviewer 2. This section now reads:  “To simultaneously assess the duration and degree of hypothermia under 36℃ during surgery, the AUC under 36℃ was calculated for each patient according to the trapezoidal rule for calculating definite integrals between each 15-minute time point (AUC=0.5*[T1-36℃+T2-36]*[15 minutes]) *where T1 = starting core body temperature and T2 = the core body temperature 15 minutes later, calculated for the duration of the surgery* [24].” |
| Line 48: Consider rephrasing "CW is associated with significantly less intraoperative hypothermia than FAW when FAW was used without prewarming" to "CW is more effective than FAW at reducing intraoperative hypothermia, especially when FAW is used without prewarming." | Modified as suggested. |
| Line 54: Consider rephrasing "PH is associated with increased blood loss, increased time to emerge from anesthesia, prolonged hospitalization, surgical site infections, and general thermal discomfort" to "PH is associated with various adverse outcomes, including increased blood loss, prolonged emergence time, extended hospital stay, surgical site infections, and patient discomfort." | Modified as suggested. |
| Line 61: The phrase "is there is no superiority" should be corrected to "there is no superiority." | Modified as suggested. |
| Line 69: Consider rephrasing "Recently, conductive warming (CW) devices, which conduct heat from a thermal blanket directly to the patient, have become popular" to "Recently, conductive warming (CW) devices have gained popularity due to their ability to directly transfer heat to the patient." | Modified as suggested. |
| Line 71: The phrase "CW devices have variable efficiency, but may be more efficient than FAW" could be rephrased to "While CW devices can vary in efficiency, they may offer superior heat transfer compared to FAW." | Modified as suggested. |
| Line 73: Consider rephrasing "Studies comparing CW and FAW efficacy have been inconclusive" to "Studies comparing CW and FAW have yielded mixed results." | Modified as suggested. |
| Line 272: Consider rephrasing "we assessed the association of four warming strategies" to "we compared the effectiveness of four warming strategies." | Modified as suggested. |
| Line 276: Consider rephrasing "CW/CW was associated with 51% less hypothermia than NAPW/FAW" to "CW/CW resulted in 51% less hypothermia compared to NAPW/FAW." | Modified as suggested. |
| Line 281: The phrase "No significant difference was observed in the pairwise comparisons between CW/CW and FAW/FAW, and NAPW/CW and FAW/FAW" is a bit convoluted. You could rephrase it as "No significant differences were observed between CW/CW and FAW/FAW, or between NAPW/CW and FAW/FAW." | Modified as suggested. |
| Lines 289-291: Consider combining the two sentences to improve readability. For example, "One possible reason for CW's superiority over NAPW/FAW is the use of an underbody CW mattress, which was not used with FAW, despite being standard practice." | This sentence was combined as suggested and now reads:  “One possible reason for CW’s superiority over NAPW/FAW is that an underbody CW mattress was always used with CW blankets, whereas no underbody blanket was used with FAW, which is the standard of care in our institution.” |
| Line 293: The phrase "variable device implementation, low-powered studies, and heterogeneity between studies making conclusions difficult" could be rephrased for better clarity. For instance, "variable device implementation, underpowered studies, and heterogeneity between studies, making it difficult to draw definitive conclusions." | Modified as suggested. |
| Lines 294-316: Consider breaking up the long paragraph into smaller paragraphs to improve readability. You could separate the discussion of the Cochrane review, the Joanna Briggs Institute review, and the specific studies by Harper et al. and others. | Modified as suggested. |
| To improve the clarity and impact of your manuscript, please adhere to the following:  1. Prioritize clarity and conciseness in writing.  2. Ensure strong evidence base and clear methodology.  3. Address limitations and propose future directions.  4. Proofread carefully for grammar and style consistency. | Edits have been made to improve readability, consistency, and quality of the written manuscript. |
| **Reviewer #2:** | |
| **Comments** | **Author Responses** |
| GENERAL  This is a very interesting paper. This reviewer is participating in the re-submission. | We thank the reviewer for their assessment of our manuscript. |
| SPECIFIC  I have a few minor comments. Normally, when referring to the AUC of core body temperature, it suggests accumulated heat (i.e., heat storage), so it is somewhat difficult to interpret the AUC in the context of this experiment. Please add a figure with data from a representative subject to illustrate the AUC. | We thank the reviewer for their suggestion. A new figure (Fig 1) that visually represents the AUC calculation has been added to the manuscript. |
| In the abstract, there is no explanation of T1 and T2. If possible, please state in the abstract that core body temperature during surgery was assessed using esophageal temperature. | Based on feedback from Reviewer 1, the methods section of the abstract was simplified and the specific formula was removed. A description of temperature measurement was added to the abstract:  “*Body temperature was measured using an esophageal probe.* The area under the temperature curve (AUC) below 36℃ was calculated according to the trapezoidal rule and quantified intraoperative hypothermia.” |
| In the methods section, please provide the gender ratio of the subjects in each group. The impact of body size differences has been considered, but as a precaution, please present basic physical characteristics in a table. This should include age and gender ratio. | Please note that demographic information including sex, BMI, and age by study arm is presented in Table 1. |
