# Supplementary material for: The optimal warming strategy to reduce perioperative hypothermia: A prospective randomized non-blinded clinical trial
Source: PLoS One. 2025 Jun 12;20(6):e0325954. doi: 10.1371/journal.pone.0325954 (PMC12161556; doi:10.1371/journal.pone.0325954)
Supplement: Attachment — Submitted filename: PONE-D-24-44335R1 rebuttal and point-by-point memo.docx [file pone.0325954.s006.docx]

**The Editors**

**PLOS One**

**RE:** PONE-D-24-44335R1

Dear Sir/Madam:

Thank you for your willingness to reconsider the manuscript. Below, you will find a point-by-point response to the questions from your editorial staff, as well as a full list of previous point-by-point memos from all prior revisions, in reverse chronological order. We are resubmitting the paper without changes to the body of the manuscript, since no specific need for changes was identified by the reviewers during the most recent review. We have only modified the labeling of the Y axis of Figure 1 to read “Esophageal temperature”. All requests for changes in R0 were addressed previously, documented in the chronology below. Methodological weaknesses have been disclosed in full in the manuscript. Our findings add value to perioperative care despite the shortcomings listed in the limitations section of the paper. This work concerns an under-researched area of perioperative care where little can be said with certainty about optimal patient warming methods, and this paper adds important insights to the small body of research that can help clinicians decide how best to warm up their patients.

Sincerely,

Ron Desai, DO

Associate Professor of Anesthesiology

On behalf of all authors

**From:** em.pone.0.91391d.442735ce@editorialmanager.com <em.pone.0.91391d.442735ce@editorialmanager.com> on behalf of PLOS ONE <em@editorialmanager.com>
**Sent:** Tuesday, February 4, 2025 3:51 PM
**To:** Desai, Ronak <desair@rowan.edu>
**Subject:** [EXTERNAL] PLOS ONE: Your Appeal for the manuscript (PONE-D-24-44335R1) - [EMID:ba89f0df8c12a091]

PONE-D-24-44335R1
The optimal warming strategy to reduce perioperative hypothermia: A prospective randomized non-blinded clinical trial
Dr Ronak Desai

Dear Dr. Desai,

I am writing to you regarding your appeal on the editorial decision for your submission to PLOS One above.

We have considered your appeal request and I am writing to let you know that we are willing to reconsider the manuscript. The manuscript should now be listed under the ‘Submissions Needing Revision’ in your manuscript folders in Editorial Manager.

We would be grateful if you could please revise the manuscript to respond to the comments raised by the Academic Editor and reviewers, and resubmit your manuscript files online once the necessary revisions are complete; please respond to all comments and implement any reasonable requests for revisions.

**Authors:** Please see below. In the most recent review, the reviewers had almost no specific requests for changes. Those requested have been addressed.

Please accompany this resubmission by a detailed rebuttal letter that includes a point-by-point response to the concerns raised by the reviewers and Academic Editor and details on the revisions carried out on the manuscript since its original submission.

**Authors:** Please consider this our detailed rebuttal letter. The Academic Editor did not raise any concerns in their most recent communication but simply declined to publish. In the most recent review, the reviewers did not explain which aspects of the manuscript they disagreed with specifically, or why; in fact, they were mostly positive about the manuscript. See below.

Please also submit a “Track Changes” version of the MS file in the File Inventory. You may submit some of the files provided in your appeal request if appropriate.

**Authors:** Multiple Word documents with track changes have already been submitted. Since there are no changed to the body of the manuscript, we are re-submitting those files.

We would be grateful if you could please complete the resubmission of the revised manuscript files within 45 days.

Please note that the manuscript may need to undergo further external review before a decision is made regarding publication and that given the previous history, the review process may be longer than for new submissions.

**Authors:** Understood.

Please do not hesitate to contact us if you have any queries regarding this process.

With best wishes, 

Melanie Española
Support Staff - Editorial
PLOS ONE

**From:** em.pone.0.910879.7b719b9d@editorialmanager.com <em.pone.0.910879.7b719b9d@editorialmanager.com> on behalf of PLOS ONE <em@editorialmanager.com>
**Sent:** Monday, January 27, 2025 12:20 AM
**To:** Desai, Ronak <desair@rowan.edu>
**Subject:** [EXTERNAL] PLOS ONE Decision: PONE-D-24-44335R1 - [EMID:91549163dced4592]

PONE-D-24-44335R1
The optimal warming strategy to reduce perioperative hypothermia: A prospective randomized non-blinded clinical trial
PLOS ONE

Dear Dr. Desai,

Thank you for submitting your manuscript to PLOS ONE. After careful consideration, we have decided that your manuscript does not meet our criteria for publication and must therefore be rejected.

I am sorry that we cannot be more positive on this occasion, but hope that you appreciate the reasons for this decision.

Kind regards,

Amirmohammad Khalaji
Academic Editor
PLOS ONE

**Authors’ response:** We thank the editor for the review but we do not see any specific reasons for the rejection, stated above.

Reviewers' comments:

Reviewer's Responses to Questions

**Comments to the Author**

1. If the authors have adequately addressed your comments raised in a previous round of review and you feel that this manuscript is now acceptable for publication, you may indicate that here to bypass the “Comments to the Author” section, enter your conflict of interest statement in the “Confidential to Editor” section, and submit your "Accept" recommendation.

Reviewer #1: All comments have been addressed

Reviewer #2: All comments have been addressed

**Authors:** The reviewers indicate that we have adequately addressed all comments in the previous round of reviews.

2. Is the manuscript technically sound, and do the data support the conclusions?

The manuscript must describe a technically sound piece of scientific research with data that supports the conclusions. Experiments must have been conducted rigorously, with appropriate controls, replication, and sample sizes. The conclusions must be drawn appropriately based on the data presented.

Reviewer #1: Yes

Reviewer #2: Yes

**Authors:** The reviewers indicate that this is a sound piece of scientific research.

3. Has the statistical analysis been performed appropriately and rigorously?

Reviewer #1: Yes

Reviewer #2: Yes

**Authors:** The reviewers indicate that the statistical analysis has been performed appropriately.

4. Have the authors made all data underlying the findings in their manuscript fully available?

The [PLOS Data policy](http://secure-web.cisco.com/181MPYGTG6Yae-wa2EDD4JuLLIuf0vAnLPWHPAs7uIJfGcoSDzw7gwvRjHG9hBBRE5meZ9o1EpOJF_NrgxO-aY2RZ4r4X9ZqIZPOYiHB3_d6OmMoIUaoL2p_R5bFNQHpN3dhhgCsDISZ_wgPoCHhtSZW4DkFY40BGPQK9NVqhuTcqDLioqtGz2Wwuxhl3tw0IkeBtMb80uaALHCqKJ3j4wk-EIjexeywmnRMvY1ffZD5JnqQkuiXwOK29Q2Lwt3mQNG9ouOCSQeeRnMz0cfnmZfxmqfIrU_Gzys_0_OdairCLUOuOQdKcI9ndy-i5Jb_p/http%3A%2F%2Ftrack.editorialmanager.com%2FCL0%2Fhttp%3A%252F%252Fwww.plosone.org%252Fstatic%252Fpolicies.action%2523sharing%2F1%2F010f0194a633e892-581d4635-aeb7-4707-b4f0-98a9792375db-000000%2Fbu0Fh0mXo6JeUR6_ealpimlhAf_0QlMqr1pFHtWOpUg%3D195) requires authors to make all data underlying the findings described in their manuscript fully available without restriction, with rare exception (please refer to the Data Availability Statement in the manuscript PDF file). The data should be provided as part of the manuscript or its supporting information, or deposited to a public repository. For example, in addition to summary statistics, the data points behind means, medians and variance measures should be available. If there are restrictions on publicly sharing data—e.g. participant privacy or use of data from a third party—those must be specified.

Reviewer #1: Yes

Reviewer #2: Yes

**Authors:** The reviewers indicate that the data sharing is appropriate.

5. Is the manuscript presented in an intelligible fashion and written in standard English?

PLOS ONE does not copyedit accepted manuscripts, so the language in submitted articles must be clear, correct, and unambiguous. Any typographical or grammatical errors should be corrected at revision, so please note any specific errors here.

Reviewer #1: No

Reviewer #2: (No Response)

**Authors’ response:** The authors are native speakers of English who have published well over 200 scientific papers among them. The manuscript in question has been edited at least four times for content and style, and most of the authors have participated in the writing and editing. The manuscript is definitely written in standard English and is more than intelligible. We find ourselves wondering if an erroneous checkmark might have been placed by Reviewer #1 (i.e. they meant yes, but clicked no).

6. Review Comments to the Author

Please use the space provided to explain your answers to the questions above. You may also include additional comments for the author, including concerns about dual publication, research ethics, or publication ethics. (Please upload your review as an attachment if it exceeds 20,000 characters)

**Reviewer #1:** The discussion is so short with many passages without citations that strengthen their evidence
The methodology is deficient in many essential areas

**Reviewer #2:** The authors responded to this reviewer's comments. However, the Y-axis label in Figure 1 should be the name of the temperature measured site, which is esophageal temperature.

**Authors’ response to reviewer #1:** Previous reviewers expressed reservations about methodology, which is why the manuscript was completely redone, the methods and statistical analysis were updated, and this resulted in a second submission. The changes made were outlined in a point-by-point memo and submitted along with the revised manuscript (PONE-D-24-44335). Subsequent reviews indicated that the issues brough up initially had been resolved, and there were only minor outstanding recommendations for improvements. This resulted in a revised submission from us, PONE-D-24-44335R1. We are now responding to the review of PONE-D-24-44335R1. Reviewer 1 does not state in what way the methodology is deficient. We are also unable to understand Reviewer #1’s comment regarding the discussion being short, without citations. The discussion is 4.5 double-spaced pages long, consisting of 1146 words and incorporating a total of 10 references, namely reference # 7, 12-15, 17-8, 20, and 28-29. It is incomprehensible that this should be considered a short and under-referenced discussion. The only passages that do not contain references are the ones summarizing our findings.

**Authors’ response to reviewer #2:** We thank the reviewer for this observation. The label of the Y axis can easily be changed to “esophageal temperature”.

7. PLOS authors have the option to publish the peer review history of their article ([what does this mean?](https://secure-web.cisco.com/1WChfoEZm-CZw4baXcPFnbACwF_A--jV5E7JW1DBbG75UlZaCLW_5GTJF8SXbe3mPi1zpoUPq4iSWQAzSW2QXGQ2HUkmPQ263YwbUHQZtW1MGEU4i3gASdsBf2f-Ce307CA5WT0pfxChDLxAiz4kwNNBs4Aw0-Izo1dom0A7c9U3M15Wa3Umy406kSn6nbLb2hemo3kAO7mFzUDmc8qjUBt7YmcXs7fYbXMg68ugG0EkMjgRWZb0vIDVU48JIS2QXuU2QEOJNVq7pNT-uopi2POrtazMhQ1mSt75hS5e-IWCvzmHbpoixEmuW7abJYhcW/https%3A%2F%2Ftrack.editorialmanager.com%2FCL0%2Fhttps%3A%252F%252Fjournals.plos.org%252Fplosone%252Fs%252Feditorial-and-peer-review-process%2523loc-peer-review-history%2F1%2F010f0194a633e892-581d4635-aeb7-4707-b4f0-98a9792375db-000000%2F0ECMdwznJZdHY9XiIsnxqCxd_N_7OYgapT6dEy8pZXM%3D195)). If published, this will include your full peer review and any attached files.


If you choose “no”, your identity will remain anonymous but your review may still be made public.


**Do you want your identity to be public for this peer review?** For information about this choice, including consent withdrawal, please see our [Privacy Policy](https://secure-web.cisco.com/1zjlpyDJjXovEM_ceqauD-XzRtDkprL80WSuBeqvVUCekZxCJTWYQQdhnPw41vmefT9CzCftO9seIKQjn69cznZP2bE-kUxf5y36kpYIvoODo4DF_D9upyiEArf-7wkHKRzG9gPuge8of17cd56MJrq1IviM0pbDeFdihUQUhhgp-kpDHnd_4loNJiezfFJ1kWd41hU1CQLjIyJAK50NwssNYabxIx5jRgq50M6gqic0NO8f45c-GpSCbQLTHXido_atKQQu_Ly1XJb3BAdeOTivZuXUTgwTaSx1u4P8eH3jetMoKt4aYboBlMBN0W9HY/https%3A%2F%2Ftrack.editorialmanager.com%2FCL0%2Fhttps%3A%252F%252Fwww.plos.org%252Fprivacy-policy%2F1%2F010f0194a633e892-581d4635-aeb7-4707-b4f0-98a9792375db-000000%2FZ9reMdFw5ykmjLw1G332hzM1SbE8q2F8lQONLKa6Thc%3D195).

Reviewer #1: No

Reviewer #2: No

[NOTE: If reviewer comments were submitted as an attachment file, they will be attached to this email and accessible via the submission site. Please log into your account, locate the manuscript record, and check for the action link "View Attachments". If this link does not appear, there are no attachment files.]

- - - - -
For journal use only: PONEDEC3

Ronak Desai, DO

Vice Chairman, Clinical Affairs, Department of Anesthesiology

Cooper University Hospital

1 Cooper Plaza

Camden, NJ 08103

T +1 (856) 342-2000

desair@rowan.edu

January 2, 2025

Amirmohammad Khalaji
Academic Editor
PLOS ONE

Dear Dr. Khalaji,

Thank you for your reviewers’ comments on our manuscript, titled “The optimal warming strategy to reduce perioperative hypothermia: a prospective randomized non-blinded clinical trial,” which was submitted to *PLOS ONE.* My coauthors and I appreciate your reviewers’ careful reading of our manuscript, their attention to detail, and their thoughtful comments and questions.

Based on their comments, my coauthors and I have made revisions to the manuscript. Per your request, we are submitting the revised manuscript with all changes highlighted. We have also summarized our revisions and responses to each comment below this letter. We think that these revisions have strengthened this manuscript and that it is now ready for reconsideration of publication in your journal.

Thank you for allowing us to resubmit this article for further consideration. We look forward to hearing from you.

Sincerely,

Ronak Desai, DO

The optimal warming strategy to reduce perioperative hypothermia: a prospective randomized non-blinded clinical trial

**Manuscript Number:** PONE-D-24-44335

| **Reviewer #1:** | |
| --- | --- |
| **Comments** | **Author Responses** |
| GENERAL  As a reviewer, I commend the authors for conducting a well-designed study to investigate the impact of different warming strategies on intraoperative hypothermia. The use of a randomized controlled trial and the objective measure of AUC below 36°C are the strengths of the study. | We thank the reviewer for their detailed assessment of our manuscript. |
| SPECIFIC  However, a few areas could be further explored: Sample Size: Consider whether the sample size was adequate to detect clinically significant differences, especially for secondary outcomes, as it was mentioned in the study limitations. | Sample size was discussed as a limitation in the methodological considerations and limitations section. This discussion was expanded to include the following sentence:  “The study had the following limitations. Study enrollments fell short of the 216 minimum needed to detect a 1.0 °C*hour difference in AUC with 80% power. The enrolled subjects were predominantly female (80%), reflecting the patient population presenting for abdominal, gynecologic, breast, plastic, and urological surgery in our hospital. Homogeneity by type of surgery was not perfect in the 10 randomization groups, as there were more patients in the abdominal and the gynecologic surgery groups than the other groups (breast, plastics/reconstructive, and urological) – see S1 Appendix - Randomization chart. *Therefore, findings from the current study would benefit from confirmation in a larger clinical trial*.” |
| Heterogeneity in Surgical Procedures: The inclusion of various surgical procedures might have influenced the results. A more homogeneous patient population could provide clearer insights. | We acknowledge that the wide range of surgical procedures may limit the interpretation of data as it decreases standardization, including a variety of exposed surgical sites which may vary in rate of heat loss. However, as noted in the manuscript, peri-operative warming is used across a wide range of surgical procedures, and studying different procedures may more closely reflect real-world use. This may make the study more interpretable across specialties, given that there were minimal limitations to inclusion based on surgical procedure type. Moreover, surgery type was included as a blocking factor in the randomization schedule to avoid imbalance across groups. |
| Non-Standardized Blanket Use: The lack of standardization in blanket use in the NAPW arms is a limitation that could affect the results | We acknowledge that the implementation of blanket use was not standardized in the NAPW group. This may introduce variability, as a patient wearing a cotton blanket may retain more heat than a patient without a cotton blanket. Patients undergoing surgery may have differences in baseline thermal comfort based on various demographic and clinical characteristics (e.g. incidence of hot flushes of peri-menopause, metabolic health, known or unknown anemia, etc). Whereas the NAPW blanket intervention was not standardized, it was the clinical standard to allow patients thermal comfort in the NAPW arms, and thus they were permitted to use or defer cotton blankets to obtain thermal comfort. Therefore, this methodology reflects real-world practice, which facilitates the generalization of results. The non-standardized use blankets in the NAPW arms is described in the methodological considerations and limitations section:  “Warmed cotton blankets were applied to patients in the NAPW arms in a non-standardized fashion, i.e. only patients who wanted them were covered with blankets, and the temperature of the blankets was not recorded.” |
| The abstract is well-structured and concise. However, consider further condensing the methods section by focusing on the key aspects of the study design, such as the randomization process, intervention details, and primary outcome measure. | The abstract was edited with a focus on further condensing content. |
| Revise the whole manuscript for any grammatical and language mistakes | Line-item edits recommended by the reviewer have been made where appropriate, or the text has been edited for the purpose of clarification. We are grateful for the reviewer’s attention to detail. Additional edits were made in a full review of the manuscript and are tracked in the highlighted version of the manuscript. |
| Line 31: Consider rephrasing "We conducted a prospective observational study" to "We conducted a prospective randomized controlled trial." | Modified as suggested. |
| Line 36: The equation for calculating AUC could be simplified or presented in a more concise format. | We added a description of T1 and T2 to the formula based on feedback from Reviewer 2. This section now reads:  “To simultaneously assess the duration and degree of hypothermia under 36℃ during surgery, the AUC under 36℃ was calculated for each patient according to the trapezoidal rule for calculating definite integrals between each 15-minute time point (AUC=0.5*[T1-36℃+T2-36]*[15 minutes]) *where T1 = starting core body temperature and T2 = the core body temperature 15 minutes later, calculated for the duration of the surgery* [24].” |
| Line 48: Consider rephrasing "CW is associated with significantly less intraoperative hypothermia than FAW when FAW was used without prewarming" to "CW is more effective than FAW at reducing intraoperative hypothermia, especially when FAW is used without prewarming." | Modified as suggested. |
| Line 54: Consider rephrasing "PH is associated with increased blood loss, increased time to emerge from anesthesia, prolonged hospitalization, surgical site infections, and general thermal discomfort" to "PH is associated with various adverse outcomes, including increased blood loss, prolonged emergence time, extended hospital stay, surgical site infections, and patient discomfort." | Modified as suggested. |
| Line 61: The phrase "is there is no superiority" should be corrected to "there is no superiority." | Modified as suggested. |
| Line 69: Consider rephrasing "Recently, conductive warming (CW) devices, which conduct heat from a thermal blanket directly to the patient, have become popular" to "Recently, conductive warming (CW) devices have gained popularity due to their ability to directly transfer heat to the patient." | Modified as suggested. |
| Line 71: The phrase "CW devices have variable efficiency, but may be more efficient than FAW" could be rephrased to "While CW devices can vary in efficiency, they may offer superior heat transfer compared to FAW." | Modified as suggested. |
| Line 73: Consider rephrasing "Studies comparing CW and FAW efficacy have been inconclusive" to "Studies comparing CW and FAW have yielded mixed results." | Modified as suggested. |
| Line 272: Consider rephrasing "we assessed the association of four warming strategies" to "we compared the effectiveness of four warming strategies." | Modified as suggested. |
| Line 276: Consider rephrasing "CW/CW was associated with 51% less hypothermia than NAPW/FAW" to "CW/CW resulted in 51% less hypothermia compared to NAPW/FAW." | Modified as suggested. |
| Line 281: The phrase "No significant difference was observed in the pairwise comparisons between CW/CW and FAW/FAW, and NAPW/CW and FAW/FAW" is a bit convoluted. You could rephrase it as "No significant differences were observed between CW/CW and FAW/FAW, or between NAPW/CW and FAW/FAW." | Modified as suggested. |
| Lines 289-291: Consider combining the two sentences to improve readability. For example, "One possible reason for CW's superiority over NAPW/FAW is the use of an underbody CW mattress, which was not used with FAW, despite being standard practice." | This sentence was combined as suggested and now reads:  “One possible reason for CW’s superiority over NAPW/FAW is that an underbody CW mattress was always used with CW blankets, whereas no underbody blanket was used with FAW, which is the standard of care in our institution.” |
| Line 293: The phrase "variable device implementation, low-powered studies, and heterogeneity between studies making conclusions difficult" could be rephrased for better clarity. For instance, "variable device implementation, underpowered studies, and heterogeneity between studies, making it difficult to draw definitive conclusions." | Modified as suggested. |
| Lines 294-316: Consider breaking up the long paragraph into smaller paragraphs to improve readability. You could separate the discussion of the Cochrane review, the Joanna Briggs Institute review, and the specific studies by Harper et al. and others. | Modified as suggested. |
| To improve the clarity and impact of your manuscript, please adhere to the following:  1. Prioritize clarity and conciseness in writing.  2. Ensure strong evidence base and clear methodology.  3. Address limitations and propose future directions.  4. Proofread carefully for grammar and style consistency. | Edits have been made to improve readability, consistency, and quality of the written manuscript. |
| **Reviewer #2:** | |
| **Comments** | **Author Responses** |
| GENERAL  This is a very interesting paper. This reviewer is participating in the re-submission. | We thank the reviewer for their assessment of our manuscript. |
| SPECIFIC  I have a few minor comments. Normally, when referring to the AUC of core body temperature, it suggests accumulated heat (i.e., heat storage), so it is somewhat difficult to interpret the AUC in the context of this experiment. Please add a figure with data from a representative subject to illustrate the AUC. | We thank the reviewer for their suggestion. A new figure (Fig 1) that visually represents the AUC calculation has been added to the manuscript. |
| In the abstract, there is no explanation of T1 and T2. If possible, please state in the abstract that core body temperature during surgery was assessed using esophageal temperature. | Based on feedback from Reviewer 1, the methods section of the abstract was simplified and the specific formula was removed. A description of temperature measurement was added to the abstract:  “*Body temperature was measured using an esophageal probe.* The area under the temperature curve (AUC) below 36℃ was calculated according to the trapezoidal rule and quantified intraoperative hypothermia.” |
| In the methods section, please provide the gender ratio of the subjects in each group. The impact of body size differences has been considered, but as a precaution, please present basic physical characteristics in a table. This should include age and gender ratio. | Please note that demographic information including sex, BMI, and age by study arm is presented in Table 1. |

August 26, 2024

Steven E. Wolf, MD
Academic Editor
PLOS ONE

Dear Dr. Wolf:

We are submitting a new, completely revised manuscript of “The Optimal Warming Strategy to Reduce Perioperative Hypothermia: A Prospective Randomized Non-Blinded Clinical Trial”. We took into account the reviewers’ recommendations from the previous submission and are sending in a point-by-point memo, in particular with respect to the statistical approach, which was redesigned. To accomplish this, we enlisted the services of John DiBato PhD, a new biostatistician at our institution who was previously not associated with the project. He reviewed the project from the ground up, including all the assumptions and the power calculation based on the main clinical outcomes of interest, and redesigned the analysis using a mixed model which, in his professional estimation, addresses all the concerns of the statistical editor from the previous submission. Dr. DiBato also edited the manuscript and has therefore been added as co-author. A detailed description of the model is submitted as supplementary material. The results were not drastically different from the initial findings and continue to be clinically relevant. We therefore feel that this study report merits a second look, and we would be delighted to work with you, especially in light of the fact that the project has already received peer review by PLOS One reviewers. We have no opposed reviewers.

All authors agree with and are responsible for the data presented. None of the authors have any conflicts of interest. All authors participated in the conceptualization, research, writing and editing of the manuscript.

We look forward to a fresh review and publication decision.

With best wishes,

_________________________

Ludmil Mitrev, MD, FASA

Associate Professor of Anesthesiology, CMSRU

Vice Chair for Research,

Department of Anesthesiology, Cooper University Healthcare

**On behalf of the authors**

**From:** em.pone.0.8bd878.d7d1f296@editorialmanager.com <em.pone.0.8bd878.d7d1f296@editorialmanager.com> on behalf of PLOS ONE <em@editorialmanager.com>
**Sent:** Tuesday, June 4, 2024 10:10 PM
**To:** Desai, Ronak <desair@rowan.edu>
**Subject:** [EXTERNAL] PLOS ONE Decision: PONE-D-24-14397 - [EMID:b5efdd1b0b06e569]

PONE-D-24-14397
The Optimal Warming Strategy to Reduce Perioperative Hypothermia: A Prospective Randomized Non-Blinded Clinical Trial
PLOS ONE

Dear Dr. Desai,

Thank you for submitting your manuscript to PLOS ONE. After careful consideration, we have decided that your manuscript does not meet our criteria for publication and must therefore be rejected.

I am sorry that we cannot be more positive on this occasion, but hope that you appreciate the reasons for this decision.

Kind regards,

Steven E. Wolf, MD
Academic Editor
PLOS ONE

Additional Editor Comments:

Academic Editor - Thank you for submitting your paper to us for review. I sent it to seven distinguished referees for comment and decision of whom three agreed to review; you will see these below. Unfortunately, the referees found some significant issues with the paper, and could not recommend publication. Most of this has to do with the statistical rendering of the results, which has some holes in it as described. For my part, I feel this is a reasonably well done study with supportable conclusions, however, the statistical issues have to be addressed fully. This might result in some significant changes in the conclusions, so the final decision was for reject but with hearty encouragement to address these issues and resubmit. It does not really fit as a major revision since the conclusions have a reasonable probability of significant change. I hope you will consider this solution, and I look forward to seeing it come back.


Reviewers' comments:

Reviewer's Responses to Questions

**Comments to the Author**

1. Is the manuscript technically sound, and do the data support the conclusions?

The manuscript must describe a technically sound piece of scientific research with data that supports the conclusions. Experiments must have been conducted rigorously, with appropriate controls, replication, and sample sizes. The conclusions must be drawn appropriately based on the data presented.

Reviewer #1: Yes

Reviewer #2: Partly

Reviewer #3: No

2. Has the statistical analysis been performed appropriately and rigorously?

Reviewer #1: Yes

Reviewer #2: I Don't Know

Reviewer #3: No

3. Have the authors made all data underlying the findings in their manuscript fully available?

The [PLOS Data policy](http://secure-web.cisco.com/1QhVL8NXuEpSb4ZBpFTF-sj2qy6laNuC_d85Bd8oTeXNhcn5y42uYKK2qG97y4wwAD6uM_Q5qgP5t5sUtgvB66x96uP3isfDA5zUwoINATKOCivkw94zSd1-wwRFhTL6elXjns1MVqWjAt3fCrcgHUKYxJItknEp3dvM-6t_Py5l3Sc9Ii2qyovXmGng0uAt8nXVeURKo_rw-5UsO8sA2_V3kFANbGZeytWXThmdy-4tYYmapAlW7HvibNlMVj0JuSRmqPOQCJZwre_7lBDkM4ZBzL39tylSq_uZEelTcLEepXCafJnD6DYzQZcAMvDG8/http%3A%2F%2Fwww.plosone.org%2Fstatic%2Fpolicies.action%23sharing) requires authors to make all data underlying the findings described in their manuscript fully available without restriction, with rare exception (please refer to the Data Availability Statement in the manuscript PDF file). The data should be provided as part of the manuscript or its supporting information, or deposited to a public repository. For example, in addition to summary statistics, the data points behind means, medians and variance measures should be available. If there are restrictions on publicly sharing data—e.g. participant privacy or use of data from a third party—those must be specified.

Reviewer #1: Yes

Reviewer #2: Yes

Reviewer #3: No

**Authors’ response re #3: We have completely revised the statistical approach and in addition to previously submitted data, we have attached the mixed model and the randomization table to the new submission.**

4. Is the manuscript presented in an intelligible fashion and written in standard English?

PLOS ONE does not copyedit accepted manuscripts, so the language in submitted articles must be clear, correct, and unambiguous. Any typographical or grammatical errors should be corrected at revision, so please note any specific errors here.

Reviewer #1: Yes

Reviewer #2: Yes

Reviewer #3: No

5. Review Comments to the Author

Reviewer #1

1. Desai and colleagues investigated the topic of perioperative hypothermia through a combination of pre- and intra-operative forced air warming and conductive warming methods. They concluded that intra-operative conductive warming (underbody plus over body cover) was superior to forced air warming when prewarming was not possible, and that intra-operative forced air warming alone is inferior to the other methods tested. However, if patients undergo prewarming then both strategies are equivalent.

The manuscript reads well, references relevant literature, and addresses a common perioperative quality metric and patient safety concern. No major weaknesses noted. One minor point is that my brief literature review revealed a couple recent systematic reviews on prevention and management of perioperative hypothermia in adult elective surgical patients, which found forced air warming to be effective and may warrant comment in the author’s discussion (Simegn G. Ann Med Surg 2021 Nov 14:72:103059 and Moola S. JBL Libr Syst Rev 2010;8(19):752-792).

**Authors’ response: Thank you for these suggestions. We have now cited two extensive reviews that are highly relevant to the discussion:**

**Madrid E, Urrutia G, Roque i Figuls M, Pardo-Hernandez H, Campos JM, Paniagua P, et al. Active body surface warming systems for preventing complications caused by inadvertent perioperative hypothermia in adults. The Cochrane database of systematic reviews. 2016;4(4):CD009016. Epub 20160421. doi: 10.1002/14651858.CD009016.pub2. PubMed PMID: 27098439; PubMed Central PMCID: PMCPMC8687605.**

**Moola S and Lockwood C. The effectiveness of strategies for the management and/or prevention of hypothermia within the adult perioperative environment: systematic review. JBI Libr Syst Rev 2010; 8: 752-792. DOI: 10.11124/01938924-201008190-00001**

The authors eloquently address how their results contrast published studies that inconclusively reported the effects of conductive warming. The study design and thorough data collection adds significant value to published work on this topic by collecting temperature measurements throughout the intra-operative time frame and using the same temperature settings regardless of device used.

I recommend publishing.

2. Areas for improvement
a. Major issues: none
b. Minor issues: consider reviewing systematic reviews noted above for potential additional discussion points

Reviewer #2:

Dear Authors,
This is a nice and interesting study. In this study, the authors have compared the effects of prewarming and intraoperative warming modes (with forced-air warming (FAW) and conductive warming (CW) methods) on the incidence and duration of hypothermia.
In this study, unlike other research, perioperative hypothermia has been evaluated by measuring the area under the curve (AUC)< 36°C.
The research contains some limitations. Failure to ensure homogeneity of patient and surgical methods and protective measures against hypothermia should be presented in the limitation section.
The statistics of the article should be re-evaluated.
I reviewed this article and have a few editing suggestions.
Sincerely
Suggestions:
1. Materials and Methods/Participants. In this prospective randomised study has evaluated patients who elective surgery under general anaesthesia. However, homogeneity could not be achieved with surgery types (major surgery or minor surgery). Were patients using medications that would affect body temperature changes excluded? The research contains some limitations. Failure to ensure homogeneity of patient and surgical methods and protective measures against hypothermia should be presented in the limitation section.

**Authors’ response: We thank the reviewer for this advice. We have added the full randomization table (“S1 Appendix - Randomization chart.xlsx”) with the types of elective surgeries enrolled and have added a comment about homogeneity in the discussion.**

2. Materials and Methods/ Randomization. (line 156-158) It is written, “Patients were contacted twice postoperatively, at 24 hours and then 30 days to assess for any complications not recorded in the electronic medical record.”. These data are not available between the primary and secondary outcoms of the study.

**Authors’ response: We have added a table to report the post-operative complications and events as supplementary material – “S3 Appendix - Secondary outcomes and complications.xlsx”.**

3. Materials and Methods/ Procedures: When was the first temperature measurement made? Please explain. Please detail other precautions to prevent perioperative temperature decrease (e.g. warming of intravenous and infusion fluids, etc.). Were the patients' basal temperatures evaluated before surgery?

**Authors’ response: We thank the reviewer for this advice. We have added this to the methods section, subsection on ‘Procedures’.**

4. “In Table 2: Regression analyses of the hypothermia AUC for each trial arm”. The purpose of the comparison between groups' linear regression test should be explained in the result section.

**Authors’ response: all tables have been completely reworked in line with the new analysis and results.**

Reviewer #3

The authors reported about the results of prospective, non-blinded, randomized single center clinical trial with a 4 arm parallel group design to explore the effect of two different ways to warming strategies (preoperative and intraoperative) with no application of preoperative warming, or FAW or CW. They concluded that intraoperative FAW alone is an inferior warming strategy compared to three other methods: CW/CW, FAW/FAW, and NAPW/CW and thus patients may benefit from the routine implementation of active warming for at least 30 minutes preoperatively. When prewarming is not feasible, intraoperative CW is a superior warming strategy compared to FAW.

In general, this is a nice study but due to major discrepancies between planning and conduct as well as planning errors, it appears to have unsurmountable weaknesses and result in unreproducible research.

To be more specific:

**Authors’ response: We thank the reviewer for the thorough review. As to the hypotheses, they were merely reworded in the original manuscript for clarity (shown in table below). However, we did a poor job of explaining that, in reality, there was only one leading hypothesis, namely that there would be a significant difference in the mean area under the curve (AUC) of temperature below 36℃ during the intraoperative period between the four combinations of warming strategies. We have now clearly stated that in the last paragraph of the introduction and have removed all references to three separate hypotheses.**

| Hypotheses as stated in the protocol | Hypotheses as stated in the original manuscript |
| --- | --- |
| 1. Active warming with CW will be non-inferior in preventing hypothermia when compared to active warming with FAW. 2. Active preoperative warming with CW or FAW will lead to a reduction in intraoperative hypothermia when compared with only intraoperative warming with CW or FAW. 3. Active preoperative warming combined with intraoperative warming using CW is a superior strategy compared to only intra-operative warming with the CW, intraoperative warming with FAW only, or pre-operative and intra-operative warming with FAW. | 1. Active intraoperative warming with CW is non-inferior to FAW in preventing intraoperative hypothermia. 2. Actively prewarming for at least 30 minutes with either CW or FAW reduces intraoperative hypothermia compared to no active prewarming (NAPW) with intraoperative CW or FAW, respectively. 3. Actively prewarming with CW combined with intraoperative CW is superior to NAPW with intraoperative CW or FAW, and is superior to prewarming and intraoperative warming with FAW. |

The author referred to 3 hypothesis (the first one differs from the initial protocol and serves as sample size justification) which may be formalized as

H01: {\mu_(CW,CW) = {\mu_( NAPW,CW)} or {\mu_(CW,CW) = {\mu_( NAPW, FAW)} or {\mu_(CW,CW) = {\mu_( FAW/FAW)}

H02: {\mu^2_(CW or FAW, CW or FAW)= {\mu^2_( NAPW, CW or FAW)}

H03: |{\mu_(CW,CW) = {\mu_( FAW/FAW)} | ≤ ∂ =1

These specific set of family of hypotheses cause a multiple testing problem, which has to be considered in the sample size justification, and analysis strategy. Moreover, the coherence between the statistical analysis model and the stratification factors of the randomization has to be considered. Both aspects are not fulfilled and thus the results and conclusion might change completely.

L150ff: Description of the randomization process: The randomization algorithm and software for generation of the randomization list is not given. The total of 10 stratification groups are not clear. In fact, the number of 10 stratification factors lead to an expected number of 18.4 patients per stratification factor level which are allocated to 4 treatment groups. This offers high predictability and thus allocation bias, in particular in a open study, depending on the randomization algorithm. Further samples size obviously does not result in a 1:1:1:1 allocation ratio.
Concerning the implementation it is not detailed, how concealment of the randomization process was achieved.

**Authors’ response: We have added the full randomization table (“S1 Appendix - Randomization chart.xlsx”) which makes the 10 stratification groups clear, and have explained in the body of the text that we used the random number function in Excel to create the table. The method of randomization concealment is also explained.**

L182: The sample size justification could not be followed for various reasons and may be wrong, resulting in a not well powered study.
First the proposed inclusion of the hypotheses, by which no adjustment of the significance level is needed, applies not to H02, as the endpoint is measured on different times. Further although the third part of H01 implies H03, the converse is not true. And of note the analysis population of H03 differs from H01. So sample size justification for H01 does not necessarily apply to the other ones.
The effect size stated for H01 declaring a difference of 1 as clinically relevant with the ST of 0.3 to 0.6 results in huge effect sizes of 1.67 to 3.3 resulting in rather small (2-7) sample sizes. This explains that the sample size justification could not be followed.
And, it is rather critical, to argue, that the non-inferiority margin has the same value, than the expected clinically relevant difference.
Calculation of adjustment for dropout rate is 4x40/0.85=188.23~189
**Authors’ response: a new sample size calculation was done based on a null hypothesis of equal means among the 4 treatment arms. This is clearly explained in the methods section. It yielded a slightly larger sample size of 216 for the study.**

L199: Measurement method of temperature should be given. Note according to the protocol different methods are used for pre/post and intraoperative measurement.

**Authors’ response: the temperature measurement method is clearly stated in the ‘Procedures’ section under ‘Methods’.**

L214: Statistical analysis is only partly according to the protocol and even there not appropriate.
The Shapiro Wilk's pretest needs to be included in a multiple testing decision rule framework. Corresponding type one error adjustment is needed. This test is superfluous in RCT's.
The statistical test on the main hypothesis does not follow the planned one. The statistical model deviates from the one used for sample size justification and the randomization process. Thus the power requirement is not given.
By the way, the stratification factor surgical approach has 5 and not 2 levels.
**Authors’ response: the statistical approach was completely redone and this is explained in detail in the new manuscript, as well as in ‘S2 Appendix - Mixed model.docx’.**

L232: Enrollment period is missing.

**Authors’ response: the enrollment period is now clearly stated under ‘Ethics approval and registration’ under ‘Methods’ (towards the end of the paragraph).**

L240: Statistical test in table 1 should be deleted as test on baseline characteristics are not meaningful in RCTs.

**Authors’ response: Table 1 has been completely redone.**

L243: Test results with application of the primary hypotheses should be stated explicitly.

**Authors’ response: this has been done in the new manuscript. The primary hypothesis and test results are stated clearly.**

L253: p-values need to be adjusted for multiple comparisons. Note regression coefficient do correspond to differences of means. These descriptive measures are informative here.
**Authors’ response: the statistical approach was completely redone and this is explained in detail in the new manuscript, as well as in ‘S2 Appendix - Mixed model.docx’.**

L350: Limitation section with discussion on the impact of non-blinding, allocation bias, missing observations etc on the study results need to be included.

**Authors’ response: this has been done. We have an expanded and updated limitations section under ‘Discussion’: ‘Methodological considerations and limitations.’**
