# Supplementary material for: The optimal warming strategy to reduce perioperative hypothermia: A prospective randomized non-blinded clinical trial
Source: PLoS One. 2025 Jun 12;20(6):e0325954. doi: 10.1371/journal.pone.0325954 (PMC12161556; doi:10.1371/journal.pone.0325954)
Supplement: Attachment — Submitted filename: Desai et al response to reviewers_R3.docx [file pone.0325954.s007.docx]

The optimal warming strategy to reduce perioperative hypothermia: a prospective randomized non-blinded clinical trial

**Manuscript Number:** PONE-D-24-44335R2

The authors should report in the description of Table 1 of the main manuscript how the data are presented in the table (values and values in brackets).

**Author Responses**

We apologize for this oversight and table 1 has been corrected.

Considering table 1, surgical approach is not similar in all groups; could the authors comment on this point?

**Author Responses**

Thank you for the comment. While some numerical differences existed in the proportions of patients who underwent open vs laparoscopic surgery across the four warming treatment arms, this difference was not found to be nominally significant (*P* > 0.05) on post-hoc testing of the distribution using a chi-squared test.

Could the authors report the mean duration of surgery in each group?

**Author Responses**

Mean duration of surgery has been added to Table 1.

The planned sample size was not reached and this is an important limitation that could reduce the strength of conclusions. The authors should emphasize this point in the conclusion.

**Author Responses**

Thank you for the suggestion, the Conclusion section on p19 has been modified to include a statement regarding this limitation (new text italicized):

“Our results suggest that CW is superior to FAW at reducing intraoperative hypothermia when FAW is used without prewarming. This refers to CW applied simultaneously as an underbody mattress and over-the-body cover. When patients can be actively prewarmed, CW/CW and FAW/FAW showed no difference in their ability to maintain normothermia. We conclude that CW may be routinely employed for perioperative warming both in the preoperative and intraoperative setting. *This study was limited by not meeting its predefined sample size for enrollment.”*

**Reviewer #2:**

**Comments**

**Author Responses**

GENERAL

This study evaluated the effectiveness of four different combinations of preoperative and intraoperative warming by comparing them to reduce perioperative hypothermia. The study design was rigorous, but some areas for improvement remain.

**Author Responses**

We thank the reviewer for their assessment.

SPECIFIC

In the inclusion criteria, it is stated that the expected duration of the operation is more than 90 minutes and less than 240 minutes. This criterion is not supported by the literature, and it is hoped that the authors will add relevant evidence to enhance the rationality and scientific validity of the inclusion criteria.

**Author Responses**

We thank the reviewer for their suggestion. However, when designing the study, we did not find a unified approach in terms of this inclusion criterion in the literature. For example, one previous randomized controlled trial included surgeries between 30-120 minutes in duration (Perl et al. *Minerva Anesthesiol* 2014;80:436-443), whereas another included surgeries 1-6 hours (Lau et al. *Can J Anaesth* 2018;65:1029-1040). We selected the expected duration of 90-240 minutes because, based on our clinical experience, it would allow for any hypothermia to occur while limiting the influence of any substantial outliers in very long surgical cases. The following was added to the Methods section on p6:

“Patients scheduled for surgery were screened for eligibility. Inclusion criteria were elective surgery under general anesthesia projected to last greater than 90 minutes but less than 240 minutes. *The expected duration of 90-240 minutes was selected because, based on clinical experience, it would allow for any hypothermia to occur while limiting the influence of any substantial outliers in very long surgical cases.*”

The frequency of intraoperative temperature measurement using the esophageal probe placed after induction of anesthesia every 15 minutes was not supported in the literature. Since the frequency of temperature monitoring has a significant impact on the accuracy of the study results, it is recommended that the authors review and supplement the literature.

**Author Responses**

In response, the following was added to the Methods section on p7:

“*Consistent with a previous randomized controlled trial on warming strategies [24],* patient temperature was measured (to the tenth degree Celsius) every 15 minutes intraoperatively using an esophageal probe placed after induction of anesthesia. IV fluids were not warmed. Room temperature was measured every 15 minutes using an Elitech RC-5 temperature logger (Elitech Technology Inc., San Jose, CA). Patients were contacted twice postoperatively, at 24 hours and then 30 days to assess for any complications not recorded in the electronic medical record.”

Considering that different types of surgeries may have different impacts on patients' temperature changes, has this study considered subgroup analyses of different types of surgeries to explore in more depth the effects of warming strategies under various types of surgeries?

**Author Responses**

We thank the reviewer for the suggestion. As described in the Methods section, stratified random sampling was employed to ensure equal distribution of surgery type across warming treatment arms. While this ensured an investigation in a sample of surgical patients broadly representative of varying surgical approaches in a large hospital, studies on individual surgery subtypes are likely more representative of those individual surgical populations. Subgroup analysis by surgery subtype in the present study would be inappropriate due to the limited sample size in individual surgical groups. To highlight this important point for readers the following point has been added to the Limitations section on p18:

“The study had the following limitations. Study enrollments fell short of the 216 minimum needed to detect a 1.0 °C*hour difference in AUC with 80% power. The enrolled subjects were predominantly female (80%), reflecting the patient population presenting for abdominal, gynecologic, breast, plastic, and urological surgery in our hospital. Homogeneity by type of surgery was not perfect in the 10 randomization groups, as there were more patients in the abdominal and the gynecologic surgery groups than the other groups (breast, plastics/reconstructive, and urological) – see S1 Appendix - Randomization chart. Therefore, findings from the current study would benefit from confirmation in a larger clinical trial. *Additionally, studies in individual surgical subtypes would likely be more representative of those individual populations.”*
